# Development and evaluation of a parent advisory group to inform a research program for knowledge translation in child health

**DOI:** 10.1186/s40900-021-00280-3

**Published:** 2021-06-07

**Authors:** Lisa Hartling, Sarah A. Elliott, Kelli Buckreus, Julie Leung, Shannon D. Scott

**Affiliations:** 1 Department of Pediatrics, Faculty of Medicine & Dentistry, 4-472 Edmonton Clinic Health Academy, University of Alberta, 11405-87 Avenue, Edmonton, Alberta T6G 1C9 Canada; 2 Faculty of Nursing, University of Alberta, Edmonton, Canada; 3 Pediatric Parent Advisory Group, University of Alberta, Edmonton, Canada

**Keywords:** Parents, Advisory group, Patient engagement, Patient-oriented research, Child health, Knowledge translation, Knowledge mobilization, Evaluation

## Abstract

**Background:**

In response to a growing movement to involve patients and community stakeholders in health research, we established a parent advisory group in 2016. The group meets regularly to act as advisors and partners for our research program. The purpose of this paper is to describe our experiences establishing the group, and results from parent evaluations.

**Methods:**

We contacted 191 organizations to recruit parents and caregivers of children who wanted to contribute to child health research. Our initial goal was to recruit 8 to 10 parents who would meet regularly (approximately 8 times per year). We conducted an online baseline survey of members after the first two meetings to understand motivations for participating and early experiences. Sixteen months later we conducted another online survey to identify what was going well and areas for improvement.

**Results:**

Twelve parents initially joined the group. The baseline survey (*n* = 9 complete) identified motivations for participation: wanting a patient/family voice in health research; personal experience accessing health system for child’s care; wanting to improve healthcare communications. Concerns about participation included: having sufficient time to attend meetings; whether contributions would be worthwhile; and uncertainty about how the group’s input would be used in practice. Parents identified aspects that were working well: opportunity to provide constructive feedback; diversity among parents involved; well-run and organized meetings (agenda and materials sent prior to meeting, skilled facilitation, adequate time for discussion). Items parents identified as not working well were: fluctuating attendance; not knowing others in the group; challenges if attending remotely. At follow-up, there were seven active members. The follow-up survey (*n* = 5 complete) identified positive feedback related to group dynamics (e.g., collegial, everyone participates) and organization of meetings. Suggestions for improvement included increasing membership, regular attendance, and providing adequate information/context to allow meaningful input.

**Conclusions:**

Our experience establishing a parent advisory group and evaluation of the group by parent members have yielded tremendous insights around involving parents and patient proxies in health research. The parent advisory group is a dynamic entity requiring ongoing communication between researchers and members. Effective means of evaluating engagement is essential to ensure it is meaningful. Dedicated time, funding and resources are required for success.

**Supplementary Information:**

The online version contains supplementary material available at 10.1186/s40900-021-00280-3.

## Plain English summary

In 2016 we developed a parent advisory group to inform our research program in knowledge translation (i.e., sharing research in accessible ways to inform decision-making) for child health. The group provides input from a parent perspective. We conducted an online baseline survey of parent members and a follow-up online survey 16 months after the group had started. The surveys helped us understand reasons for parents’ participation, what they thought was working well with the group, and areas for improvement. Parents valued the opportunity to provide constructive feedback on research processes and outputs. They felt the meetings were well-organized and the group was open and welcoming. Parents felt that regular and ongoing communication with the researchers was critical for meaningful engagement. To this end, funding to support a dedicated coordinator was considered essential. Parents appreciated that the researchers organized the group around parents’ needs in terms of timing of meetings (evenings) and reimbursement for expenses to attend meetings (travel, childcare). Parents considered this type of group to be unique, particularly as it supports a research program over the long-term rather than for specific projects, and because of the relative maturity in terms of clearly defining group purpose, structure, and engagement approach. The ongoing involvement allows for benefits in terms of building relationships, providing many and varied opportunities to interact, and allowing parents to see how their input is implemented. Finally, an evaluation mechanism with communication of results and a commitment to implement findings is considered critical. Our parent advisory group can provide a model for other researchers or research organizations.

**Supplementary Information:**

The online version contains supplementary material available at 10.1186/s40900-021-00280-3.

## Background

Over the past 10 to 15 years, there has been a growing movement to involve patients and community stakeholders in health research. This has been motivated by increasing recognition that patient involvement can enhance research processes, make research outputs more relevant, optimize knowledge translation (KT; i.e., communication and uptake of research by healthcare providers, health systems, and patients to inform decision-making), and ultimately improve health outcomes. In 2009/2010 the Canadian Institutes for Health Research incorporated a focus on patient-oriented research (POR) into their strategic planning, recognizing POR as the cornerstone of evidence-informed decision-making. This led to the Strategy for Patient Oriented Research (SPOR) with a significant investment of funds and multiple initiatives country-wide [1]. Similar directions and investments for POR have occurred in other jurisdictions, [1] including the United States, [2] United Kingdom, [3] Australia, [4, 5] and multiple countries in Europe [6, 7].

**Fig. 1:**
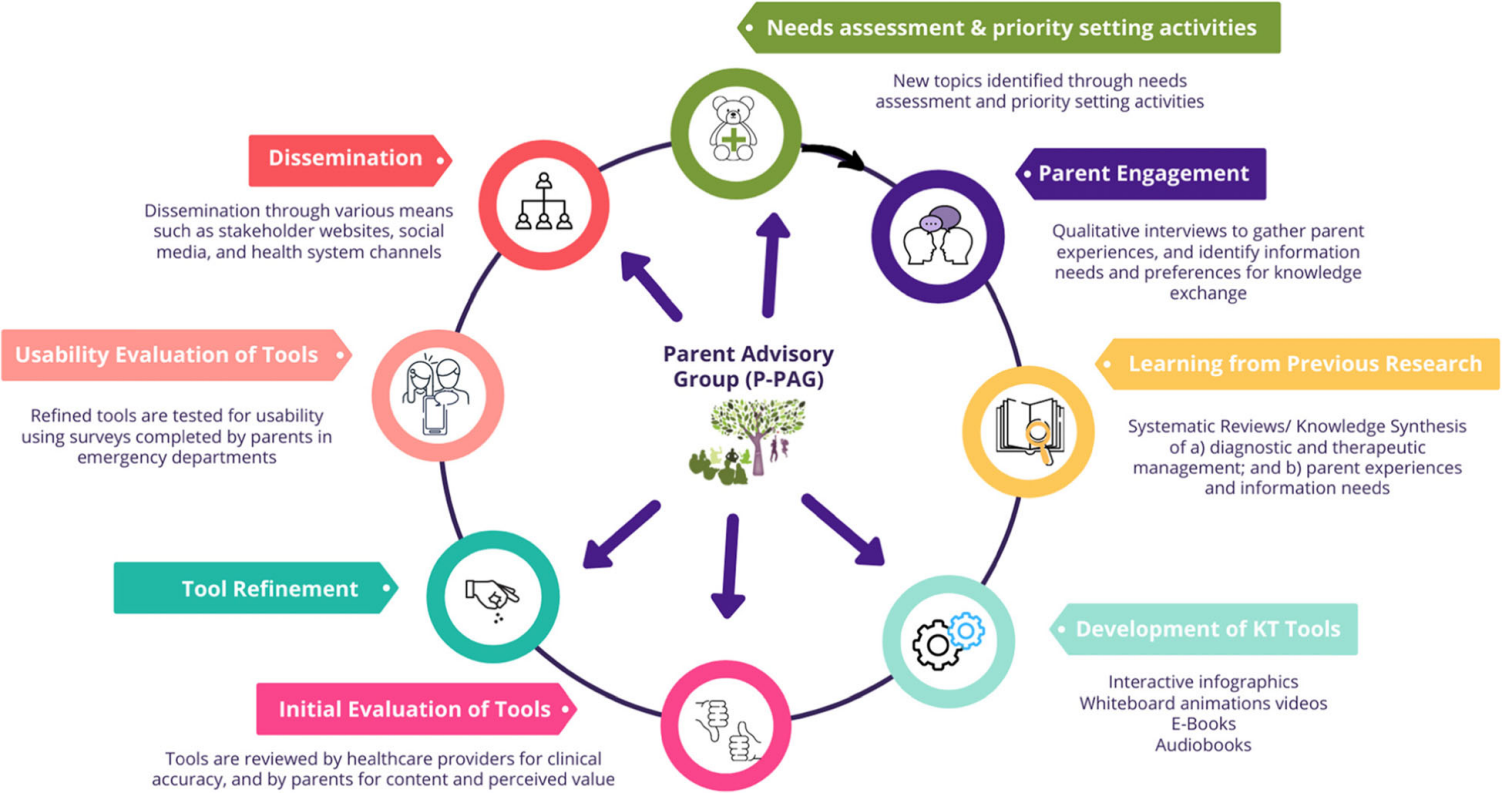
Process for the development of our knowledge translation tools for parents

Key to improving health outcomes in children is actively engaging parents in decisions about their child’s health. Our research aims to develop, evaluate, and disseminate KT tools (e.g., infographics, whiteboard animation videos) to increase parent confidence and knowledge, and support decision-making for common acute conditions. We began with a national needs assessment of healthcare providers and parents to identify priority conditions, health information seeking practices, and information needs [8–11]. We use a structured process involving seven key stages and multiple methods to develop KT tools (Fig. 1). For each condition, we: conduct systematic reviews to synthesize evidence on therapeutic management, and parent experiences and information needs; undertake qualitative interviews with parents to further understand their experiences and information needs; develop prototypes for KT tools that are vetted by parents for content and perceived value, and by healthcare providers for clinical accuracy; and, conduct usability testing of the final tools. We currently have 20 tools on a variety of conditions (publicly available at echokt.ca/tools).

Involving parents in the development of our KT tools is essential to ensure they meet parent needs. Initially we recruited parents on an ad hoc basis to identify their experiences and information needs, and test KT tool usability. As our research program matured, we had the opportunity to create an ongoing parent advisory group. Our objective was to establish a group that would meet regularly to advise (provide advice, guidance and knowledge from a parent perspective) and partner (co-develop content for the KT tools) on our various research activities [12].

The purpose of this paper is to describe our experiences establishing the Pediatric Parent Advisory Group (P-PAG) and results from initial evaluations of the group by its members. In particular we wanted to share our experiences and learnings about: how recruitment was initially done; why parents wanted to get involved; challenges and facilitators to maintaining membership and regular attendance; required time, effort, and resources to develop and maintain the group; and, processes and value of formal evaluations to ensure parents have a mechanism to provide feedback about the group, its operations, and whether they feel meaningfully engaged.

## Methods

We invited parents and caregivers from across our province (Alberta, Canada, which is under a single health system) to join the P-PAG. Eligibility criteria included: a parent, grandparent or legal guardian of a child (less than 18 years); wanting to contribute to child health research; willingness to work collaboratively with a group; and able to attend regular meetings in-person, by telephone, or online. We indicated that the intent of group membership was to contribute to research aimed at improving children’s health outcomes, and that we would seek their advice on: digital KT tool prototypes; effective means of working with parents and families as “researchers”; and, identifying child health research priorities.

In April and May 2016, we contacted 191 local/regional organizations, including parent organizations comprising subsidiary groups, branches or stakeholders (such as the local school board and local university-community partnership). Information was circulated via newsletters and listservs. The communications office at our provincial health system (Alberta Health Services) placed posters at clinical sites. We also shared information through our research groups’ social media accounts and personal contacts; and, posters were placed around our university campus.

We asked parents to email us describing their interests and experience. Parents who demonstrated general eligibility were invited to a meeting which was conducted in person when possible or by telephone. Typically, two representatives of the research groups met with each interested parent. Two representatives met so that we could provide more comprehensive context and information regarding the researchers’ programs of research and so selection was based on the appraisal of more than one research team member. During the meeting we discussed the group in more detail, e.g., purpose, structure, meeting frequency, member expectations. Together the researchers and the parent decided whether membership would be a good fit.

### Structure

The P-PAG was to be self-governed with a Chair serving a 1-year term and selected by the group from among its members. The Chair works with the researchers to develop meeting agendas and runs the meetings. The coordinator (research staff) is responsible for sending out the agenda and any meeting materials, and any other communications between meetings. During the first year, we held an initial meeting in June 2016 then bimonthly from September 2016 to May 2017. The meetings occurred during a weekday evening, and typically lasted 1.5 h. Parents were reimbursed for costs associated with attending meetings (e.g., parking, childcare). Snacks were provided at each meeting. Parents were offered a gift card (approximately Cdn$25) twice a year in recognition of their participation.

### Evaluation

We pre-planned evaluations to: 1) understand parent expectations and motivations for joining; 2) offer an anonymous mechanism for members to provide feedback; 3) ensure the orientation sessions adequately prepared members to participate (Supplementary Material 1); and 4) ensure members felt their voice was heard and respected.

We planned for online surveys (Supplementary Material 2) near the start of members joining the group (baseline survey) and near the end of each year of operations (follow-up survey) (Appendix 1 and 2, respectively). An email with the link to the voluntary surveys as well as instructions was sent to members. Consent was implied by survey completion and submission. No identifying information was collected and all responses were anonymous.

### Data analysis

Data were analyzed using descriptive statistics. Text responses were analyzed by content analysis.

### Ethics approval

We communicated that participation in the evaluations was optional and was not required as part of group membership. The evaluation plan was approved by the University of Alberta’s Health Research Ethics Board (Pro00066847).

## Results

### Group membership

We initially had 12 members (8 mothers, 3 fathers, 1 grandfather); four members identified as immigrants to Canada. Members had experience in the following work sectors: health system; business; government; non-governmental organization; education; media. Members learned about the P-PAG from various sources, e.g., Community and University Partnership, Edmonton Public School Board, Stollery Family-Centered Care Network, Cerebral Palsy Association of Alberta, Gateway Association, Edmonton Immigrant Services Association, and personal contacts. There were few applicants who did not meet the eligibility criteria. There was only one that was not accepted as the applicant had extensive prior and ongoing research experience and their motivation for joining related to that rather than as a parent/family member. Several others chose not to join after meeting and understanding the purpose, effort, and commitment required. As a key objective in its first year, the P-PAG discussed a process for selection of a chair from among its members; a chair was elected during the first year (in February 2017).

### Meeting content

**Fig. 2:**
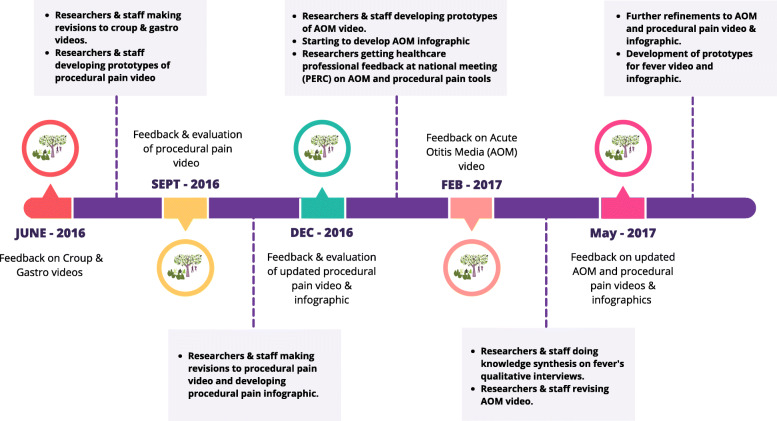
Overview of parent advisory group input on knowledge translation tools

Figure 2 provides an overview of the meetings and activities by the researchers in between meetings. At the first meeting, we reviewed and parents signed a confidentiality agreement indicating that discussions or information shared at the meetings would not be communicated outside the group. We reviewed orientation materials (i.e., terms of reference, See Supplementary Material 1), and the lead researchers provided an overview of their research programs. We discussed tasks for future meetings (e.g., communication methods and frequency, meeting logistics, evaluation plan, review of terms of reference and standard operating procedures, criteria for membership). Also at the first meeting we conducted a focus group where parents provided input on two KT tools on croup and gastroenteritis. Subsequent meetings during the first year were held in September and December 2016, then February and May 2017. Parents provided feedback on several KT tools including prototypes for whiteboard animation videos and interactive infographics about procedural pain and acute otitis media; revised versions were shared at subsequent meetings. Two topics involved work by graduate students who also attended the meetings to present and lead discussions. Parents gave feedback about the content of the tools, draft scripts, character styles and video storyboards. Parents were also involved in a priority setting project [13].

### Evaluation

We conducted a baseline survey after our second meeting in September 2016 (*n* = 9, 75% completed). Common reasons for joining were: wanting to be involved and have a patient/family voice in health research; having personal experience accessing the health system for their child’s care; wanting to improve healthcare communications. Concerns about membership included: having sufficient time to attend meetings; whether contributions would be worthwhile (i.e., whether their input would be used and have an impact on health outcomes); and uncertainty about how the researchers’ work fit into the larger “health machine”. Parents reported on what was working well: opportunity to provide constructive feedback; good variety in terms of parents’ backgrounds, culture, experience, gender and perspectives; meetings were well-run and organized; group was open and welcoming; there appeared to be genuine desire on the part of the researchers for parent input. Items that parents identified as not working well were: fluctuating attendance; challenges if attending remotely; and feeling shy or not knowing others in the group.

Table 1 presents the baseline results related to parents’ experiences with the group. Parents agreed or strongly agreed with 8 of 14 items. Several items showed less agreement: for one item one parent somewhat disagreed (I feel my views are respected and valued); and for two items there were several parents who neither agreed nor disagreed (I think P-PAG will make a difference in pediatric research; I am confident that P-PAG will yield the desired outcomes). Table 2 presents results of parents’ initial impressions of P-PAG’s leadership and management. Parents responded ideal or very good for three of the six items. One parent was neutral for each of two items about the amount of time for discussion and follow-up, and communications after each meeting. One parent thought meeting frequency was less than ideal, recommending more frequent meetings. One concern was about the motivations of the researchers—that is, whether P-PAG was itself a research project and whether it would serve its “announced purpose”. This parent wanted to know if the researchers had objective means of assessing P-PAG’s effectiveness, beyond asking members if they felt good about the experience.

**Table 1:** Results from Baseline Survey: Parent Experience (*n* = 9)

	Strongly Agree n (%)	Somewhat Agree n (%)	Neither Agree nor Disagree n (%)	Somewhat Disagree n (%)	Strongly Disagree n (%)
I understand the purpose of P-PAG.	8 (89)	1 (11)	–	–	–
I understand my own role on the P-PAG.	8 (89)	1 (11)	–	–	–
The supports I need to participate P-PAG are available (e.g., travel costs, preparation for meetings).	7 (78)	1 (11)	1 (11)	–	–
I have enough information to contribute to the topics being discussed.	8 (89)	1 (11)	–	–	–
I feel confident contributing to the discussions.	7 (78)	2 (22)	–	–	–
I have the opportunity to express my opinions when I have something to say.	7 (78)	2 (22)	–	–	–
I feel that my views are heard.	8 (89)	1 (11)	–	–	–
I feel that my views are respected and valued.	6 (67)	1 (11)	1 (11)	1 (11)	–
It is clear when and why my opinions are being sought.	7 (78)	2 (22)	–	–	–
If there are differences of opinion or disagreements, they are handled appropriately.	4 (44)	4 (44)	1 (11)	–	–
I feel P-PAG is a good use of my time.	6 (67)	2 (22)	1 (11)	–	–
If we needed members, I would be comfortable recommending P-PAG to a colleague or friend.	6 (67)	3 (33)	–	–	–
I think P-PAG will make a difference in Pediatric Research.	3 (33)	3 (33)	3 (33)	–	–
I am confident that P-PAG will yield the desired outcomes.	3 (33)	3 (33)	3 (33)	–	–

**Table 2:** Results from Baseline Survey: P-PAG Leadership and Management (*n* = 9)

	Ideal n (%)	Very Good n (%)	Neutral n (%)	Less than ideal n (%)	Unacceptable n (%)
The overall scope of P-PAG (what we are trying to achieve and the boundaries of the group).	3 (33)	6 (67)	–	–	–
The frequency of the meetings.	4 (44)	4 (44)	–	1 (11)	–
The amount of time during meetings to discuss items.	3 (33)	5 (55)	1 (11)	–	–
The overall leadership or management of the meetings.	2 (22)	7 (78)	–	–	–
The amount of time provided to review all communication and materials.	5 (55)	4 (44)	–	–	–
Follow-up and communication after each meeting.	4 (44)	4 (44)	1 (11)	–	–

In October 2017 (after 16 months), we administered a follow-up survey. At that time there were seven active members and five (71%) responded. Four had no concerns with the group, while one member expressed concern about level and consistency of group membership and attendance. When asked if staff could do anything differently, one parent suggested a team activity outside of formal meetings, and another suggested recruiting more members. What parents thought was working well related to group dynamics (e.g., collegial tone to conversations, group interacts well, everyone gets a chance to speak, researchers provide just the right amount of input without taking over) and organization of meetings. Suggestions for improvements included increasing numbers and regular attendance.

Table 3 presents the follow-up survey results about parent experience with the group. Parents strongly or somewhat agreed with 11 items. For the remaining four items, one or two parents neither agreed nor disagreed: the supports needed to participate are available; enough information is provided to contribute to discussions; the group will make a difference in pediatric research; and confidence that the group will yield the desired outcomes. Table 4 provides results regarding leadership and management, which were more variable. Two parents thought the frequency of the meetings was less than ideal, and for most of the items one parent chose ‘neutral’. Parents commented on sometimes needing more information or context to provide meaningful input (e.g., about the specific health issue, broader dissemination plans). Parents suggested inviting more researchers to the meetings, to present on their work and obtain input from the group. One parent suggested that researchers seek input earlier during an initiative rather than when the study is complete and parent input is focused on KT. All but one parent (who provided no response) indicated that they would continue partaking in the P-PAG. Table 5 provides a summary of results from the follow-up survey.

**Table 3:** Results from Follow-up Survey: Parent Experience (n = 5)

	Strongly Agree n (%)	Somewhat Agree n (%)	Neither Agree nor Disagree n (%)	Somewhat Disagree n (%)	Strongly Disagree n (%)
I understand the purpose of P-PAG.	4 (80)	1 (20)	–	–	–
I understand my own role on the P-PAG.	3 (60)	2 (40)	–	–	–
The supports I need to participate P-PAG are available (e.g., travel costs, preparation for meetings).	2 (40)	2 (40)	1 (20)	–	–
I am given enough information to contribute to the topics being discussed.	2 (40)	2 (40)	1 (20)	–	–
I feel confident contributing to the discussions.	4 (80)	1 (20)	–	–	–
I have the opportunity to express my opinions when I have something to say.	3 (60)	2 (40)	–	–	–
I feel that my views are heard.	3 (60)	2 (40)	–	–	–
I feel that my views are respected and valued.	4 (80)	1 (20)			–
It is clear when and why my opinions are being sought.	2 (40)	3 (60)	–	–	–
If there are differences of opinion or disagreements, they are handled appropriately.	3 (60)	2 (40)	–	–	–
I am satisfied with the decision-making process.	3 (60)	2 (40)	–		
I feel P-PAG is a good use of my time.	5 (100)	–	–	–	–
If we needed members, I would be comfortable recommending P-PAG to a colleague or friend.	3 (60)	2 (40)	–	–	–
I think P-PAG will make a difference in Pediatric Research.	1 (20)	3 (60)	1 (20)	–	–
I am confident that P-PAG will yield the desired outcomes.	2 (40)	1 (20)	2 (40)	–	–

**Table 4:** Results from Follow-up Survey: P-PAG Leadership and Management (n = 5)

	Ideal n (%)	Very Good n (%)	Neutral n (%)	Less than ideal n (%)	Unacceptable n (%)
The overall scope of P-PAG (what we are trying to achieve and the boundaries of the group).	–	4 (80)	1 (20)	–	–
The frequency of the meetings.	–	2 (40)	1 (20)	2 (40)	–
The amount of time during meetings to discuss items.	1 (20)	3 (60)	1 (20)	–	–
The overall leadership or management of the meetings.	1 (20)	3 (60)	1 (20)	–	–
The amount of time provided to review all communication and materials.	1 (20)	3 (60)	1 (20)	–	–
Follow-up and communication after each meeting.	3 (60)	2 (40)	–	–	–

**Table 5:** Summary of Results from Follow-up Survey

Good things about the group	Things the group could work on
• Welcoming environment • Diverse group • Range of opinions and points of view • Respectful and constructive communication • Positive group dynamics • Organized, well-facilitated • Overall, the purpose of the P-PAG and role of the parents was clear	• Add in team-building ice breaker activities • Reinforce that all opinions are valued, respected and heard • Ensure everyone has the opportunity to express opinions • Build a committed core that attends every meeting • Ensure appropriate technology available for members participating remotely • Consider more frequent meetings

## Discussion

Our experience establishing a parent advisory group for our research program and evaluation by group members have been extremely valuable and have yielded tremendous insights around involving parents or patient proxies in health research, including how to engage them meaningfully and the logistics and resources involved. Table 6 summarizes the researchers’ experience and key lessons learned from the first year of running the P-PAG. While there is much interest and activity to engage patients in health research, there is limited literature describing experiences and providing evidence on the development and engagement process, particularly for a group providing long-term support and commitment. Our hope in sharing our experiences and resources (e.g., evaluations, terms of reference) is that it will help others who are developing or maintaining an advisory group. In particular, it is critical that those undertaking a similar initiative understand the resources required, as well as the ongoing and iterative work involved (e.g., repeated recruitment efforts, reasons patients may or may not participate, mechanisms to ensure continued involvement, regular opportunity for member feedback through formal evaluations, etc.). Our experience, which offers many considerations, is particular to parents and acute childhood conditions within a specific jurisdiction and may not be generalizable to other contexts (e.g., chronic conditions, other patient populations, other regions where healthcare systems and practices may differ). For example, levels of interest, engagement, and commitment may be different for groups that focus on specific conditions, particularly those that are chronic.

**Table 6:** Summary of researchers’ experience with the pediatric parent advisory group

Item	Reflections
Group is a dynamic entity	Iterative, ongoing feedback process between researchers and group members
Time commitments	Ongoing effort to foster relationship with parents Needing to ensure that research staff are available to attend evening meetings
Dedicated funding and resources	For parents: compensation for time, transportation, parking, child care, food For staffing: dedicated time (with appropriate skill sets) to set up, facilitate and regularly communicate with group members; conduct regular evaluations involving quantitative and qualitative research methods
Communications	Clear, and with follow-up regarding outcomes from each project/activity
Bigger picture	Regular communication about where they fit in the larger program objectives
Ethics approval	Does that change the conversation with parents, e.g., if they become research subjects
Training	What (if any) training should be provided, how often, and should it be repeated regularly. Does training change the member, nature of the group, perspective, and nature of the feedback or input members provide.

Developing and maintaining the group has been an iterative process. Our initial intent was to have a core group of 8–10 parents, with the number based on recommendations for size of focus groups [14]. However, we learned that not all parents could attend each meeting; therefore, we considered a larger membership, anticipating that we could expect on average 8–10 parents to attend any given meeting. While we started with 12 parents, only 7 continued after the first year. A recurring theme from parents’ responses to the evaluation surveys was the need and desire for more members and regular participation. When parents discontinued membership, the coordinator asked the parent if they were willing to share the reason. As this information was not gathered with ethics approval, we have not reported details here; however, one of the main reasons for discontinuation was limited time.

We sought to involve parents from different backgrounds aiming for diversity in education, sex, gender, family structure, culture, and socioeconomic status. While our initial group membership was quite diverse, we had challenges maintaining diversity with respect to some factors. For instance, we involved grandparents, fathers and single parents, and members from different cultural groups; however, the majority were mothers with university educations and from two-parent, middle to high income households. We continue to reflect on means to increase diversity while recognizing the barriers to participation that some individuals may face (e.g., interest in subject matter, time, childcare, travel, technology). Key areas where we want to increase diversity relate to type of parent (e.g., fathers, single parents) and socioeconomic status (e.g., income, education, health literacy).

Coordinating the group requires a substantial investment including time, specific skill sets (e.g., group facilitation, stakeholder engagement) and other resources [15]. We found that a dedicated full-time coordinator is essential for regular communications with the group, organizing meetings, implementing membership strategies, and conducting regular evaluations. Effective coordination requires skills in engagement, group facilitation, and quantitative and qualitative research methods. Dedicated funding is essential for the coordinator role, meeting costs (e.g., food, materials), and parents’ costs (e.g., parking, childcare). We regularly offered parents gifts (e.g., gift cards) as a token of appreciation. Since we started the group, compensation guidelines for patient engagement have been produced [16–18]. There are potential barriers to implementing these guidelines including the level of funding required and administrative and financial support to disburse payments (and tax slips if required). Further, compensation may have tax implications for participants with associated time and effort on their part [19]. Finally, compensation may change the nature, and possibly the quality, of the input parents/patients provide. For example, compensation may increase the quality of feedback from a motivated patient partner because it may encourage greater focus, increased time investment, and more thoughtful engagement on subject matter that may be difficult to relate to, uninteresting, or challenging to get through. Alternatively, if participation is solely motivated by reimbursement, they may be less engaged and provide less meaningful input. These are key considerations for our group and the field of patient engagement in general.

We learned that regular communication was essential to effective operations. However, we wanted to balance ongoing communication with not overwhelming or being a burden to parents. In addition to regular meetings, we sent a maximum of three emails in between meetings. One email provided the agenda, time and location of the next meeting, and any materials for discussion. A key piece of information that parents requested was follow-up on discussion items. For example, when they gave feedback on KT tool prototypes, they also wanted to hear about other feedback (e.g., from healthcare providers), how we integrated feedback, what changes were made, and how the final products appeared. They also wanted to know where they fit into the larger research program. Further, they wanted to know how or whether their input had any impact on the health system or health services provided, and ultimately child health.

One concern was about the evaluation process itself. We planned for regular evaluations to ensure we had an anonymous feedback mechanism so that we could improve the group; as well, we wanted to share our experiences to inform other researchers and more generally the fields of patient engagement and patient-oriented research. In order to report our findings, we had to obtain ethics approval and enroll parents as participants in the evaluation. This changed the role of our parent members from research advisors to research participants; one parent questioned whether the P-PAG was simply a research exercise. This parent also questioned how we would assess the group’s effectiveness. This is a challenge yet key priority for patient engagement, that is, to identify effective means of assessing engagement and ensure the purpose for engaging patients is realized [15, 20].

A final consideration in developing the group was how much training to offer the members. We asked the group members if they wanted educational sessions integrated into the meetings, e.g., about research methods, etc. The group members felt that this would change the nature of the group and the perspectives and feedback that we originally sought. Based on this input, we did not provide structured training or educational sessions; however, over time, the group members did become more knowledgeable. This provides benefits as they are more aware of processes, how their feedback is used, and how to be more purposeful in their involvement and feedback they provide; however, it also brings a challenge as they may no longer represent a ‘typical’ parent. This is an important consideration for those developing similar groups; one strategy we are implementing is to regularly recruit and add new members to the group.

While we hope that our experiences are helpful to those who want to undertake a similar initiative of developing an advisory group or involving patient partners in research activities, the information we have gathered will also guide our future work. We plan to maintain our parent advisory group. We recognize the need for regular recruitment to ensure we maintain sufficient members. We are also implementing suggestions from parents to increase engagement, including providing regular feedback about how the parent input was used. Our original plans were to involve parents as they are a proxy for our patient group of young children; this was driven by our initial priority topics which largely affect children under 5 years and where the end-user of our KT tools is parents (i.e., parents are making the healthcare decisions). However, as we identify other priority topics, we are investigating the possibility of a youth advisory group. As a first step, we undertook an environmental scan to identify whether there are existing groups that we can access instead of establishing our own.

## Conclusions

This research identified parents’ motivations and expectations for engaging with researchers and being involved in the translation and dissemination of pediatric health research. Parents provided essential information on how to improve the structure, function and processes associated with the group. Engaging patients in the design, conduct, analysis and translation of research is a growing area, however, little evidence is available on the best way to engage and ensure the experience is beneficial for all stakeholders involved. We trust that our experience and reflections will contribute to this critical area of patient engagement, and offer a starting point for others wanting to establish an advisory group, to ensure the purpose for engaging patients is realized.

### Supplementary Information

**Additional file 1.**

**Additional file 2.**

## Data Availability

The datasets used and/or analysed during the current study are available from the corresponding author on reasonable request.

## Appendix 1

### Baseline Survey

#### Introduction

Thank you very much for your time and commitment to the Pediatric Parent Advisory Group (P-PAG). The organizations leading the P-PAG (the ECHO and ARCHE research groups) are interested in evaluation to ensure that the initiative meets its desired outcomes, that advisory group members have a positive experience, and to inform future planning. We would appreciate it if you took a few moments to complete this survey about your experience on the P-PAG. Please answer this survey from the perspective of when you first became involved in the P-PAG. Only aggregate responses will be included in the summary report (i.e., the responses of the whole group) and no individual respondents will be identified in the summary. The evaluation is being conducted via an online survey and your submission of the survey implies consent to participation in this evaluation. This larger evaluation will occur twice in the next year: now (early on in the process) and again after one year with shorter surveys being provided after every second P-PAG meeting to allow for quick course adjustments.

Please answer these questions from the perspective of when you first became involved in P-PAG.

1. Thinking back to when you were first invited to participate in the parent advisory group, what was the biggest factor in your decision to say yes? Please elaborate.
2. Thinking back to when you were first invited to participate in the parent advisory group, did you have any reservations, concerns or hesitation at that time about the merits of this group or whether it would be a worthwhile endeavor for you? Please elaborate.
3. At this early evaluation period, do you still have those initial concerns or reservations, or any new concerns, about the parent advisory group? Please elaborate.
4. At this early juncture for you personally what, if anything, would you say is working well for this group?
5. At this early juncture, for you personally what, if anything, would you say is NOT working well for this group?
6. Please indicate your level of agreement or disagreement with the following statements regarding your experiences so far with the parent advisory group:

**Table:**

	Strongly Agree	Somewhat Agree	Neither Agree nor Disagree	Somewhat Disagree	Strongly Disagree
I understand the purpose of P-PAG.					
I understand my own role on the P-PAG.					
The supports I need to participate P-PAG are available (e.g., travel costs, preparation for meetings).					
I have enough information to contribute to the topics being discussed.					
I feel confident contributing to the discussions.					
I have the opportunity to express my opinions when I have something to say.					
I feel that my views are heard.					
I feel that my views are respected and valued.					
It is clear when and why my opinions are being sought.					
If there are differences of opinion or disagreements, they are handled appropriately.					
I feel P-PAG is a good use of my time.					
If we needed members, I would be comfortable recommending P-PAG to a colleague or friend.					
I think P-PAG will make a difference in Pediatric Research.					
I am confident that P-PAG will yield the desired outcomes.					

If desired, please elaborate on any of the items above or the reasons behind your assessment.

#### P-PAG leadership and management

7. Please assess the following aspects of P-PAG and its management. Space is provided after this question if you’d like to elaborate further on any of your responses to these items:

**Table:**

	Ideal	Very Good	Neutral	Less than ideal	Unacceptable
The overall scope of P-PAG (what we are trying to achieve and the boundaries of the group).					
The frequency of the meetings.					
The amount of time during meetings to discuss items.					
The overall leadership or management of the meetings.					
The amount of time provided to review all communication and materials.					
Follow-up and communication after each meeting.					

As desired, please elaborate further on any of the items above or your reasons behind your scoring.

8. Have you noticed any challenges aspects of working with researchers? Please elaborate, including what could alleviate these issues.
9. Any other comments or suggestions?

## Appendix 2

### Follow-up Survey

#### Introduction

Thank you very much for your time and commitment to the first year of P-PAG. It is amazing how quickly the first year has flown by. We planned an evaluation at the 1-year anniversary of P-PAG and we would appreciate it if you took a few moments to complete this survey about your experiences over the last year. There is no identifying information collected by these surveys and only aggregate responses will be included in the summary report (i.e., the responses of the whole group). This evaluation is being conducted via an online survey and your submission of the survey implies consent to participate.

1. At this evaluation period, do you still have any of those initial concerns or reservations identified in the first survey, or any new concerns, about P-PAG? Please elaborate.
2. Thinking back over the last year, is there anything that the support staff could have done differently or is there any additional information that could have been provided to improve your experience?
3. What, if anything, would you say is working well?
4. What, if anything, would you say is NOT working well?
5. Please indicate your level of agreement or disagreement with the following statements regarding your experiences:

**Table:**

	Strongly Agree	Somewhat Agree	Neither Agree nor Disagree	Somewhat Disagree	Strongly Disagree
I understand the purpose of this P-PAG.					
I understand my own role on P-PAG.					
The supports I need to participate in P-PAG are available (e.g., travel costs, preparation for meetings).					
I have enough information to contribute to the topics being discussed.					
I feel confident contributing to the discussions.					
I have the opportunity to express my opinions when I have something to say.					
I feel that my views are heard.					
I feel that my views are respected and valued.					
It is clear when and why my opinions are being sought.					
If there are differences of opinion or disagreements, they are handled appropriately.					
I am satisfied with the decision-making process.					
I feel P-PAG is a good use of my time.					
If we needed additional members, I would be comfortable recommending P-PAG to a colleague or friend.					
I think P-PAG will make a difference in pediatric research.					
I am confident that P-PAG will yield the desired outcomes.					

If desired, please elaborate on any of the items above or the reasons behind your assessment.

#### P-PAG leadership and management

6. Please assess the following aspects of P-PAG and its management. Space is provided after this question if you’d like to elaborate further on any of your responses to these items:

**Table:**

	Ideal	Very Good	Neutral	Less than ideal	Unacceptable
The overall scope of P-PAG (what we are trying to achieve and the boundaries of the group.					
The frequency of the meetings.					
The amount of time during meetings to discuss items.					
The overall leadership or management of the meetings.					
The amount of time provided to review all communication and materials.					
Follow-up and communication after each meeting.					

As desired, please elaborate further on any of the items above or your reasons behind your scoring.

7. Have you noticed any challenges aspects of working with researchers? Please elaborate, including what could alleviate these issues.
8. Based on your experience over the last year, would you like to continue participation on P-PAG? Please elaborate.
9. Any other comments or suggestions?
10. Would you willing to participate in an interview to elaborate on your experiences as a P-PAG member?

## Abbreviations

KT: knowledge translation
POR: patient-oriented research
P-PAG: Pediatric Parent Advisory Group
