# Supplementary material for: Development and evaluation of a parent advisory group to inform a research program for knowledge translation in child health
Source: Res Involv Engagem. 2021 Jun 7;7:38. doi: 10.1186/s40900-021-00280-3 (PMC8186233; doi:10.1186/s40900-021-00280-3)
Supplement: Supplementary file 1 — Additional file 1. [file 40900_2021_280_MOESM1_ESM.docx]

## Supplementary Material 1

**TERMS OF REFERENCE
Pediatric Parents’ Advisory Group (P-PAG)
to
ECHO Research (Translating Evidence in Child Health to enhance Outcomes) Alberta Research Centre for Health Evidence (ARCHE) and Cochrane Child Health1**

**1.0 Summary**

There is a shift underway in health care towards patient-centred care, wherein patients and their families actively engage in health care decision-making. Essential to advancing this mission in child health and simultaneously improving child health outcomes is ensuring that parents/families can access the essential, often complex health information they need to meaningfully participate in health care decisions. Towards this objective, the ECHO Research (Faculty of Nursing), ARCHE and Cochrane Child Health (Department of Pediatrics) programs at the University of Alberta aim to engage parents/families in developing processes and resources for connecting parents/families with research-based information on children’s healthcare. These processes include knowledge synthesis (collecting and integrating the most up-to-date information from research and clinical experts to meet the unique needs of parents/families) and knowledge translation (developing, refining and evaluating information resources and tools for sharing this information with parents/families). The focus will be on commonly occurring pediatric conditions for which emergency department care is commonly sought.

Each year millions of children visit emergency departments (EDs) for minor medical complaints, such as sore throats, fevers, earaches, bone fractures and concussions. This results in considerable financial and emotional costs for families and for the health care system. Information resources for parents/families that incorporate research-based knowledge on children’s healthcare can potentially reduce healthcare utilization and high hospitalization rates. The goal of ECHO Research, ARCHE and Cochrane Child Health is to improve how research-based knowledge about children’s healthcare is developed and shared with parents/families, to aid them in caring for their sick children at home and understanding when emergency department care should appropriately be sought.

ECHO Research, ARCHE and Cochrane Child Health, will provide support for the Pediatric Parents’ Advisory Group (P-PAG) and its volunteer members.

ECHO Research, ARCHE and Cochrane Child Health participate in the broader Translating Emergency Knowledge for Kids (TREKK) program that is funded by the Government of Canada’s Networks of Centres of Excellence.

**2.0 Goals and Objectives**

The P-PAG will be advisory to the ECHO Research, ARCHE and Cochrane Child Health programs. The main role of P-PAG Members is to provide parent perspectives and to reflect on information from ECHO Research, ARCHE and Cochrane Child Health about caring for sick children and seeking emergency care. Members will not be asked to provide feedback about their own personal/ family experience with children in emergency departments.

1 Cochrane Child Health is part of the broader Cochrane organization, an independent global network of physicians, researchers, patients and others across 120 countries. Cochrane works to turn the evidence generated through research into useful information for making everyday decisions about health. *Cochrane takes its name from* ***Dr. Archibald Leman Cochrane*** *(1909–1988) who advocated for the use of randomized control trials to make medicine and health services more effective and efficient.*

Goals for 2016-2017:

1. Document the process of formulating the P-PAG and its procedures;
2. Select a Chair / Co-Chairs;
3. Support child health researchers, where appropriate and feasible, to ensure their research is meaningful/appropriate for parents and families;
4. Evaluate and provide feedback on ECHO Research, ARCHE and Cochrane Child Health deliverables as they become available (e.g. videos for parents, updates to the program websites);
5. Provide thought and discussion on how P-PAG can support the future objectives of ECHO Research, ARCHE and Cochrane Child Health;
6. Identify opportunities to connect or collaborate with other groups or initiatives that will support the goals and objectives of ECHO Research, ARCHE and Cochrane Child Health.

**3.0 Membership**

P-PAG membership should consist of:

- 10-to-15 parent volunteer members
  - Parents, grandparents or legal guardians of children under 18 years of age
  - Volunteers with some engagement experience in the health care system, if possible;
  - People who are open, curious, and who want to participate to improve the health system;
  - Volunteers who are interested in building something new and who are able to provide feedback about the piloting of P-PAG to make improvements to process, etc. along the way;
- Administrative support from ECHO Research, ARCHE and Cochrane Child Health.

Terms and expectations of P-PAG members

- Term: One-year term, from September to August, with the opportunity for additional year(s) for those who are interested;
- The P-PAG will meet 6-10 times per year in-person or via teleconference. Members will be expected to attend a minimum of four (4) in-person meetings each year;
- In-person meetings will take place on weekends or evenings and will include meals/refreshments;
- Members will carry out assigned work outside of meetings and will be expected to meet deadlines for input;
- Staff supporting the P-PAG will provide feedback to members within 2 to 3 weeks of receiving their work/input;
- Members will be asked to provide feedback on P-PAG meetings, processes, support, structure, etc.;
- New members will be provided an orientation, by a current P-PAG member and support staff, prior to their first meeting.

Support

- Members of the P-PAG will be volunteers of the University of Alberta;
- ECHO Research, ARCHE and Cochrane Child Health will provide direct support for P-PAG and its Members so that they can carry out their role;
- ECHO Research, ARCHE and Cochrane Child Health will provide regular updates our their research programs and how P-PAG input has impacted their research;
- Members will have support for transportation/parking whenever needed for in-person meetings.

**4.0 Meetings and procedures**

- The P-PAG will meet 6-10 times a year in-person or via teleconference. A minimum of four (4) in- person meetings should take place each year;
- Meetings will be chaired by a parent who has participated in the P-PAG for at least one year. Two parents can act as co-chairs if this is the preferred method and will alternate the responsibility of chairing a meeting;
- In the absence of a Chair / Co-Chairs being selected, the P-PAG coordinator will temporarily serve this role;
- The Chair(s) will set meeting agendas and review requests for P-PAG research access and engagement per the P-PAG Standard Operating Procedures (SOP), with input from ECHO Research, ARCHE and Cochrane Child health administrative team.

**5.6 Voluntary research participation**

- P-PAG Members may be invited to participate directly in the research activities of ECHO Research, ARCHE and Cochrane Child Health, e.g. focus group interviews or surveys to evaluate the usability of knowledge translation resource and tool prototypes;
- An invitation to participate in research activities will be communicated to Members by the P- PAG Chair(s), and not directly by the researchers;
- Research participation will be voluntary and will involve a separate consent process;
- Research participation and other P-PAG membership activities will not be mutually exclusive;
- ECHO Research, ARCHE and/or Cochrane Child Health will provide support for research participants in carrying out these activities.
