# Supplementary material for: Development and evaluation of a parent advisory group to inform a research program for knowledge translation in child health
Source: Res Involv Engagem. 2021 Jun 7;7:38. doi: 10.1186/s40900-021-00280-3 (PMC8186233; doi:10.1186/s40900-021-00280-3)
Supplement: Supplementary file 2 — Additional file 2. [file 40900_2021_280_MOESM2_ESM.docx]

## Supplementary Material 2

**Survey Development**

The two surveys were developed using Fluidsurveys (SurveyMonkey Inc. San Mateo, California, USA), and piloted internally (n=4) among the research team to assess survey usability and technical functionality.

The open 12-page surveys consisted of a Consent Form page, Introduction page, 9 survey pages (1 question per page) and a thank-you page. Survey responses were automatically captured via the Fluidsurveys online reporting site.

All survey questions appeared in the same order for every participant. Answers were recorded as open text statements and Likert scale scores (5-point scales).

**Survey Dissemination and Process**

Information about each evaluation survey was sent by email to all members prior to conducting the evaluation. This was followed by an opportunity to discuss and ask questions at a subsequent meeting. Members were also invited to ask questions by email or telephone following the meeting.

A link to the open online surveys and corresponding Information Letter and Consent Form were then distributed to all group members via email. The surveys were hosted on the Fluidsurveys website, and only those with the survey link could complete the survey. To prevent peer or other pressure to participate, members were asked to complete the surveys on their own time. Completing the survey was voluntary, and participation in the evaluation did not affect a member’s position within the group. Group members did not receive any incentives to complete the survey.

Participants were instructed to read the Information Letter (including details about the length of time of the survey, which data were stored and where and for how long, who the investigator was, and the purpose of the study) and provide consent to participate, before filing out the survey. The survey was anonymous, and no identifying personal information was collected or stored. Participants did not have to complete all surveys questions and could review and change their answers when needed using a Back Button. Participants were also able to save their responses and then complete at a later date, as long as the survey was accessed via the initial email link.

**Survey Data collection**

The Fluidsurveys reporting website and affiliated survey account was password protected; only members of the study team could enter the site and download responses. Survey data were downloaded and the data log file cleaned in Excel.

Participation rates were calculated as the number of participants who consented and started the survey. Unique visitors were determined using IP addresses captured by the survey. Duplicate entries (identified using IP address of participant’s computer/tablet) were avoided by keeping only the most recent entry for analysis. Completion rate was calculated by the number of people who consented and submitted the survey. All data captured were used for analysis.
